# Supplementary material for: Age-based and reproductive biology of the Pacific Longnose Parrotfish Hipposcarus longiceps from Guam
Source: PeerJ. 2017 Nov 29;5:e4079. doi: 10.7717/peerj.4079 (PMC5712210; doi:10.7717/peerj.4079)
Supplement: Figure S1 — Dashed black line represents the mean change in R2 across all 6 traits, whereas dashed grey line represents the mean change in R2 across traits excluding t50 (the only trait that showed a reduction in explained variance). t50 = age at female maturity (years), L1−3 = modelled growth (in mm) from age 1 to 3 years, L50 = length at female maturity (mm), Lmax = mean maximum length (mm), L50 = length at sex change (mm), Tmax = mean maximum age (years). [file peerj-05-4079-s001.docx]

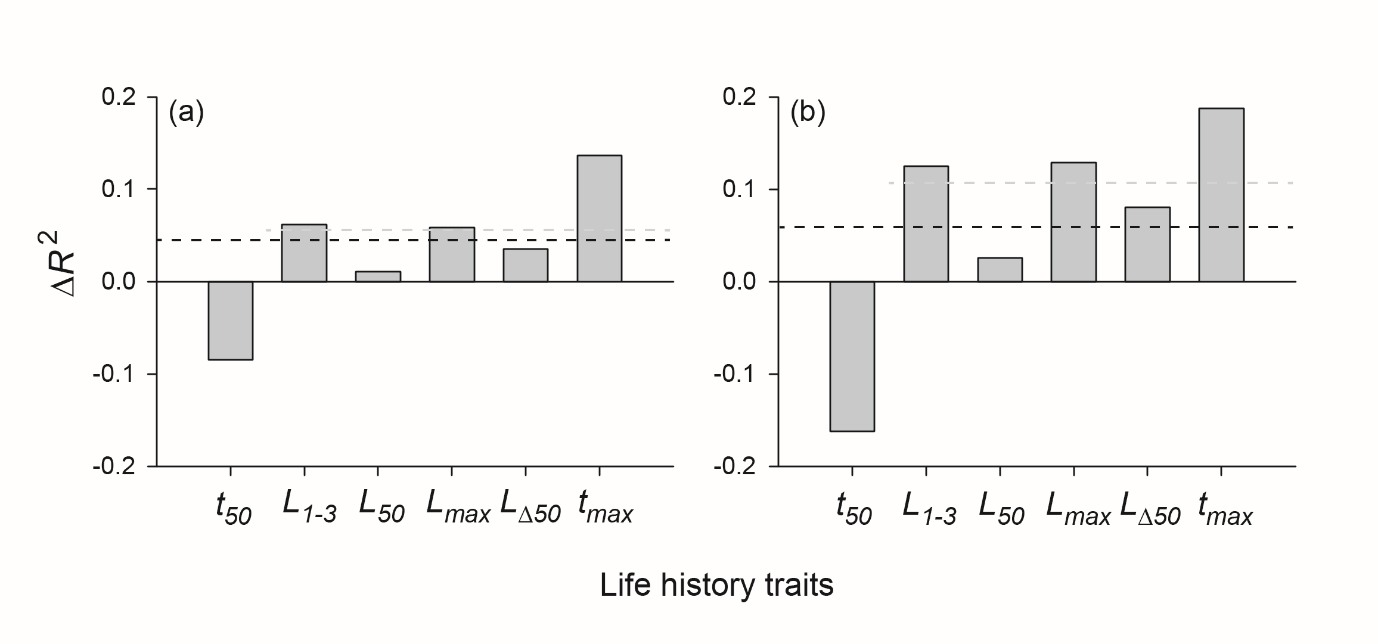


Supplementary Figure 1. Change in explained variance (*R*^2^) of models relating (a) incidence of vulnerability to overexploitation and (b) magnitude of vulnerability to overexploitation with various life-history traits across twelve parrotfish species from Guam after substitution of Guam-specific *Hipposcarus longiceps* data in lieu of data previously derived from Pohnpei, FSM (Taylor et al. 2014). Dashed black line represents the mean change in *R*^2^ across all 6 traits, whereas dashed grey line represents the mean change in *R*^2^ across traits excluding *t_50_* (the only trait that showed a reduction in explained variance). *t_50_* = age at female maturity (years), *L_1-3_* = modelled growth (in mm) from age 1 to 3 years, *L_50_* = length at female maturity (mm), *L_max_* = mean maximum length (mm), *L_∆50_* = length at sex change (mm), *T_max_* = mean maximum age (years).
